# Stainable hepatic iron in 341 African American adults at coroner/medical examiner autopsy

**DOI:** 10.1186/1472-6890-5-2

**Published:** 2005-01-10

**Authors:** James C Barton, Ronald T Acton, Asia K Richardson, Robert M Brissie

**Affiliations:** 1 Southern Iron Disorders Center, Birmingham, Alabama, USA; 2 Department of Medicine, University of Alabama at Birmingham, Birmingham, Alabama, USA; 3 Immunogenetics Program, Department of Microbiology, University of Alabama at Birmingham, Birmingham, Alabama, USA; 4 Jefferson County Coroner/Medical Examiner Office, Birmingham, Alabama, USA; 5 Division of Forensic Pathology, Department of Pathology, University of Alabama at Birmingham, Birmingham, Alabama, USA

## Abstract

**Background:**

Results of previous autopsy studies indicate that increased hepatic iron stores or hepatic iron overload is common in African Americans dying in hospitals, but there are no reports of hepatic iron content in other cohorts of African Americans.

**Methods:**

We investigated the prevalence of heavy liver iron deposition in African American adults. Using established histochemical criteria, we graded Perls' acid ferrocyanide-reactive iron in the hepatocytes and Kupffer cells of 341 consecutive African American adults who were autopsied in the coroner/medical examiner office. Heavy staining was defined as grade 3 or 4 hepatocyte iron or grade 3 Kupffer cell iron.

**Results:**

There were 254 men and 85 women (mean age ± 1 SD: 44 ± 13 y vs. 48 ± 14 y, respectively; p = 0.0255); gender was unstated or unknown in two subjects. Approximately one-third of subjects died of natural causes. Heavy staining was observed in 10.2% of men and 4.7% of women. 23 subjects had heavy hepatocyte staining only, six had heavy Kupffer cell staining only, and one had a mixed pattern of heavy staining. 15 subjects had histories of chronic alcoholism; three had heavy staining confined to hepatocytes. We analyzed the relationships of three continuous variables (age at death in years, hepatocyte iron grade, Kupffer cell iron grade) and two categorical variables (sex, cause of death (natural and non-natural causes)) in all 341 subjects using a correlation matrix with Bonferroni correction. This revealed two positive correlations: hepatocyte with Kupffer cell iron grades (p < 0.01), and male sex with hepatocyte iron grade (p < 0.05). We also analyzed the relationship of steatosis, inflammation, and fibrosis/cirrhosis in 30 subjects with heavy iron staining using a correlation matrix with Bonferroni correction. There were significant positive correlations of steatosis with inflammation (r = 0.5641; p < 0.01), and of inflammation with fibrosis/cirrhosis (r = 0.6124; p < 0.01).

**Conclusions:**

The present results confirm and extend previous observations that heavy liver iron staining is relatively common in African Americans. The pertinence of these observations to genetic and acquired causes of iron overload in African Americans is discussed.

## Background

Hepatic iron overload was detected by Perls' acid ferrocyanide staining and atomic absorption spectrometry at autopsy in more than one percent of African American adults who died in hospitals [1,2]. In one study [1], liver specimens from 326 unselected adult African Americans subjects were stained for iron; liver iron was quantified using atomic absorption spectrometry in subjects in whom increased stainable iron was observed. Four subjects (1.2%), two men and two women aged 50 to 63 years, had hepatic iron indices adjusted for previous erythrocyte transfusion that were ≥ 1.9 (range 1.9 – 5.6) [1]. In a second study [2], hepatic iron concentrations of liver tissue from autopsies in 99 African Americans were quantified. Thirty-one (31.3%) had an elevated hepatic iron concentration, including nine (9.1%) who had an hepatic iron concentration greater than twice the upper limit of normal and no evident cause of secondary iron overload [2]. These results suggest that iron overload not attributable to erythrocyte transfusion is relatively common in African Americans.

In contrast, screening programs that included cohorts of African Americans presumably representative of the general African American population identified a much lower proportion of subjects with possible iron overload [3-5] than is suggested by the results of hospital autopsy series [1,2]. These studies used an elevated transferrin saturation phenotype criterion generally regarded as the best for screening whites for *HFE*-associated hemochromatosis [3-5]. In these studies, ≤ 0.9% of African Americans adults had a positive screening result(s), and ≤ 0.09% were subsequently demonstrated to have hemochromatosis or iron overload [3-5]. Taken together, these observations suggest that previous reports of increased hepatic iron content in African Americans dying in hospital may have overestimated the prevalence of non-transfusion iron overload in African American adults in the general population, or that the ideal phenotype for screening African Americans for primary iron overload differs from that which is optimal for screening whites for *HFE*-associated hemochromatosis.

Thus, we graded Perls' acid ferrocyanide-reactive iron in the livers of 341 consecutive African American adults who underwent autopsy in the coroner/medical examiner office. We selected this population for study because such subjects are more representative of the general African American population than persons who died in hospital. We then compared these observations with autopsy results reported previously in African Americans who died in hospitals [1,2]. The pertinence of these observations to genetic and acquired causes of iron overload in African Americans is discussed.

## Methods

### Selection of study subjects

The performance of this study was approved by the Institutional Review Board of the University of Alabama at Birmingham and by the Coroner/Medical Examiner's Office of Jefferson County, Alabama. We evaluated formalin-fixed tissue obtained in 361 consecutive, unselected coroner/medical examiner autopsy cases of African-American adults (age >18 years) in Jefferson County, Alabama; deaths in all of the present cases occurred during the interval 1998 – 2002. Each subject was identified as African American by his/her previous medical histories or legal records, or by the coroner/medical examiner staff. Most autopsies were performed on the same day that the respective bodies were available for evaluation at the Coroner/Medical Examiner's Office; other autopsies were completed within ~ 18 hours. All tissues were placed directly in fixative at the time of collection at autopsy. Liver was not available or was not interpretable due to autolysis in 20 cases. Available records in each case were reviewed; age at death, sex, summary of known illnesses, and cause of death were tabulated for all cases for which liver tissue was available.

### Histologic technique, iron grading, and liver morphology

Tissues obtained at autopsy were routinely fixed in 10% neutral buffered formalin. Triplicate sections of paraffin-embedded liver were prepared. One section was stained with hematoxylin and eosin, another with Perls' acid ferrocyanide technique to demonstrate non-heme ferric iron [6], and a third with Masson trichrome technique. Appropriate positive and negative control specimens were prepared with each staining batch and reviewed.

All slides were simultaneously reviewed by three investigators, and the iron grades assigned in each case represent their consensus opinions. Hepatocellular iron was graded according to these criteria: grade 0 – no visible iron; grade 1 – iron visible in very few hepatocytes; grade 2 – iron visible in 5 – 10% of hepatocytes; grade 3 – iron visible in ≥ 40% of hepatocytes; and grade 4 – abundant iron visible in most hepatocytes [7]. Kupffer cell iron was graded according to these criteria: grade 0 – no visible iron in Kupffer cells; grade 1 – iron visible in ≤ one-third of Kupffer cells; grade 2 – iron visible in one third to ≤ two-thirds of Kupffer cells; and grade 3 – abundant iron visible in more than two-thirds of Kupffer cells [7]. Hepatocyte or Kupffer cell iron of grades 0 or 1 was defined as normal. Increased stainable iron was defined as hepatocyte and/or Kupffer cell iron grade ≥ 2 [7]. Heavy iron staining was defined as hepatocyte iron grade of 3 or 4, or Kupffer cell iron grade of 3. Steatosis, inflammation, and fibrosis/cirrhosis were assessed as described in detail elsewhere [8]; these abnormalities were graded as absent (0) or present (+).

We designated a gradient of stainable iron in hepatocytes from the periportal area decreasing towards the hepatic venule as present or absent; visualization of a gradient required hepatocyte iron staining of grade ≥ 2 [9,10]. The presence or absence of hepatic cirrhosis was determined using Masson trichrome-stained specimens as described previously [8].

### Statistical considerations

The present data set consisted of observations in 341 subjects and their respective livers. Analyses were performed with a computer spreadsheet (Excel 2000^®^, Microsoft Corp., Redmond, WA), and a statistical program (GB-Stat^® ^v. 10.0, 2003, Dynamic Microsystems, Inc., Silver Spring, MD). Descriptive data are displayed as enumerations, percentages, mean ± 1 S.D., medians, and ranges. Frequency values were compared using chi-square analysis or Fisher exact test, as appropriate. Mean values were compared using student t-test. Some data were analyzed using a correlation matrix with Bonferroni correction. Values of p < 0.05 were defined as significant.

## Results

### General characteristics of study subjects

There were 254 men (mean age 44 ± 13 y; median age 42 y; range 26 – 99 y), and 85 women (mean age 48 ± 14 y; median age 45 y; range 26 – 89 y). The mean age of men was lower than that of women (p = 0.0255). There were two subjects for whom gender was unstated or unknown. Approximately one-third of subjects died of natural causes (Table 1).

**Table 1:** Causes of death of 341 African Americans autopsied in the coroner/medical examiner office.^1^

Causes of Death	Subjects, n	Percentage
Homicide	130	38.1
Natural Cause	107	31.4
Accident	90	26.4
Unknown	7	2.1
Suicide	5	1.5
Not Stated	2	0.6

^1^Chronic alcoholism was listed for 15 subjects (the respective cause of death in each subject was ruled to be "natural cause"). Two subjects were reported to have diabetes mellitus; one had grade 3 hepatocyte and grade 1 Kupffer cell iron. No subject was reported to have iron overload, hemochromatosis, heritable or acquired forms of anemia, or treatment with erythrocyte transfusion.

### Liver iron grades

In the 341 evaluable subjects, there was a significant positive correlation of grades of stainable iron in hepatocytes and Kupffer cells across the 341 evaluable subjects (Pearson r coefficient = 0.2370; p < 0.0001). However, the mean hepatocyte iron grade was 0.83 ± 0.96; the mean Kupffer cell iron grade was 0.32 ± 0.32.

Increased hepatocyte iron grades were observed in 64 men (25.2%) and 8 women (9.4%) (p = 0.0021). Increased Kupffer cell iron grades were observed in 20 men (7.9%) and one woman (1.2%) (p = 0.0266) (Tables 2, 3).

**Table 2:** Iron grades of 341 Perls' acid ferrocyanide-stained liver sections.^1^

Grade	No. of subjects with hepatocyte staining (%)	No. of subjects with Kupffer cell staining (%)
0	164 (48.1)	270 (79.2)
1	104 (30.5)	50 (14.7)
2	49 (14.4)	14 (4.1)
3	21 (6.2)	7 (2.1)
4	3 (0.9)	not available^1^

^1^The present grading system does not include Kupffer cell iron staining greater than grade 3. In all subjects, the mean hepatocyte grade (± 1 SD) was 0.83 ± 0.96; the mean Kupffer cell grade was 0.32 ± 0.31. A gradient of stainable iron in hepatocytes from the periportal area decreasing towards the hepatic venule was observed in 56 subjects.

**Table 3:** Histologic findings in 30 African American subjects with heavy liver iron staining^1^

Age, years	Sex	Hepatocyte iron grade	Kupffer cell iron grade	Steatosis	Inflammation	Fibrosis/cirrhosis
26	M	3	1	0	0	0
27	M	1	3	0	0	0
28	M	3	0	0	0	0
29	M	3	0	0	0	0
30	M	3	1	0	0	0
34	M	3	0	0	0	0
34	M	0	3	0	0	0
37	M	3	2	+	+	0
37	M	3	1	+	+	0
39	M	3	1	0	0	0
39	M	3	1	0	+	+
40	M	2	3	+	+	0
42	M	3	0	0	0	0
43	M	3	0	0	0	0
44	M	4	1	0	+	0
44	M	4	0	0	+	+^2^
46	M	3	0	0	+	0
49	M	3	0	0	0	0
50	M	1	3	0	+	0
52	M	3	0	+	+	+
55	M	3	0	0	0	0
59	M	3	0	+	+	0
59	M	0	3	0	+	+
63	M	3	1	+	+	+
67	M	2	3	0	+	+
91	M	3	0	+	+	+^2^
33	F	3	3	0	0	0
50	F	4	0	0	+	+^2^
51	F	3	0	+	+	+
54	F	3	0	0	0	0

^1 ^Heavy iron staining was defined as hepatocyte iron grade of 3 or 4, or Kupffer cell iron grade of 3. Steatosis, inflammation, and fibrosis/cirrhosis were assessed as described in detail elsewhere [8]; these abnormalities were graded as absent (0) or present (+).

^2 ^These subjects had hepatic cirrhosis [8].

**Figure 1:**
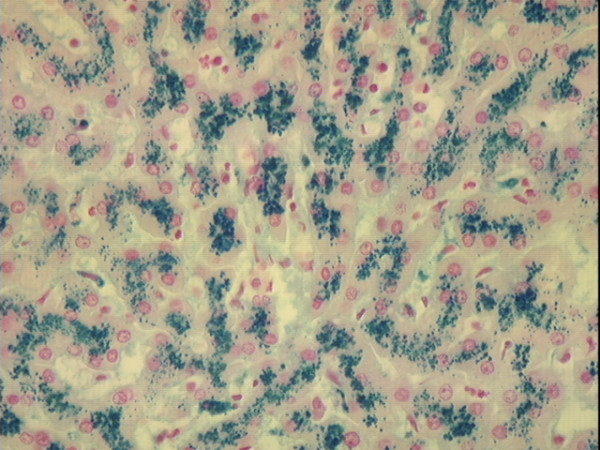
**Photomicrograph of non-cirrhotic liver stained with Perls' technique. **Liver of a 44 year-old African American man who died of pneumonia. There is a predominance of iron staining (grade 4) in hepatocytes. Original magnification 40×.

**Figure 2:**
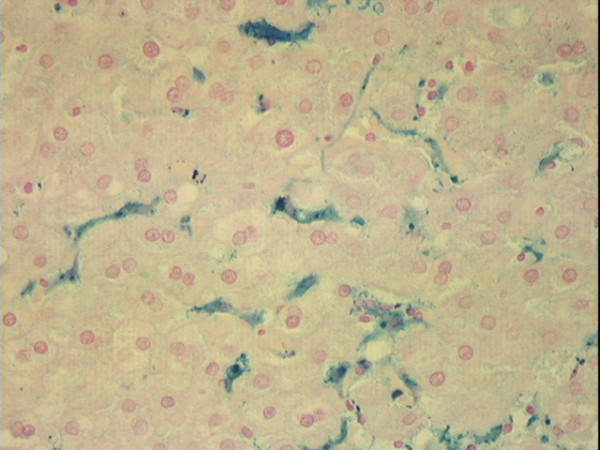
**Photomicrograph of non-cirrhotic liver stained with Perls' technique. **Liver of a 34 year-old African American man who died of homicide. There is a predominance of iron staining (grade 3) in Kupffer cells; there is faint diffuse staining of hepatocytes (grade 1). Original magnification 40×.

**Figure 3:**
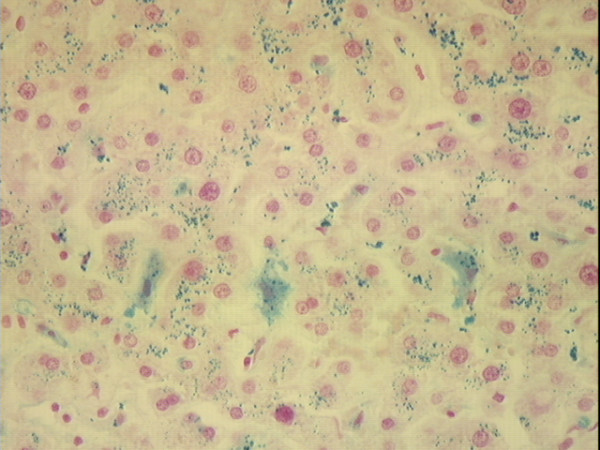
**Photomicrograph of non-cirrhotic liver stained with Perls' technique. **Liver of a 33 year-old African American woman who died of accidental trauma. There is heavy iron staining in hepatocytes (grade 3) and Kupffer cells (grade 3). Original magnification 40×.

Heavy iron staining was observed in 30 subjects (8.8%), including 10.2% of men and 4.7% of women (p = 0.1202). The mean age of men and women with heavy iron staining was similar: 45 ± 15 y and 47 ± 9 y (p = 0.7389). 24 subjects (7.1%) had grade 3 or 4 hepatocyte iron; of these, one also had grade 3 Kupffer cell iron. Seven subjects (2.1%) had grade 3 Kupffer cell iron; of these, one also had grade 3 hepatocyte iron. Altogether, 23 subjects had heavy iron staining in hepatocytes only (Fig. 1), six subjects had heavy iron staining in Kupffer cells only (Fig. 2), and one subject had a mixed pattern of heavy hepatocyte and Kupffer cell iron staining (Fig. 3). The causes of death of the 30 subjects who had heavy iron staining were similar to those of subjects with lower iron grades (data not shown).

We analyzed the relationships of three continuous variables (age at death in years, hepatocyte iron grade, and Kupffer cell iron grade) and two categorical variables (sex, cause of death (natural and non-natural causes)) using a correlation matrix with Bonferroni correction. This revealed two significant positive correlations: hepatocyte iron grade with Kupffer cell iron grade (p < 0.01), and male sex with hepatocyte iron grade (p < 0.05).

### Histologic findings in 30 subjects with heavy iron staining

These subjects were comprised of 26 men and 4 women (Table 3). Hepatic steatosis was observed in eight subjects (26.7%), hepatic inflammation was observed in 16 subjects (53.3%), and hepatic fibrosis or cirrhosis was observed in nine subjects (30.0%). However, 14 subjects (46.7%) did not have steatosis or inflammation; none of these 14 subjects had fibrosis/cirrhosis.

We analyzed the relationship of hepatocyte and Kupffer cell iron grades, steatosis, inflammation, and fibrosis/cirrhosis in these 30 subjects (Table 3) using a correlation matrix with Bonferroni correction. There was a significant negative correlation of hepatocyte and Kupffer cell iron grades (r = -0.7368; p < 0.01). There was a significant positive correlation of steatosis with inflammation (r = 0.5641; p < 0.01). There was a significant positive correlation of inflammation with fibrosis/cirrhosis (r = 0.6124; p < 0.01).

### Subjects with chronic alcoholism

Fifteen subjects had histories of chronic alcoholism; three of these had heavy liver iron staining. In each case, iron staining was confined to hepatocytes. The percentage of subjects with heavy iron staining was similar in 15 subjects reported to have chronic alcoholism and in 326 subjects not reported to have chronic alcoholism (20.0% vs. 8.3%; p = 0.1356).

**Figure 4:**
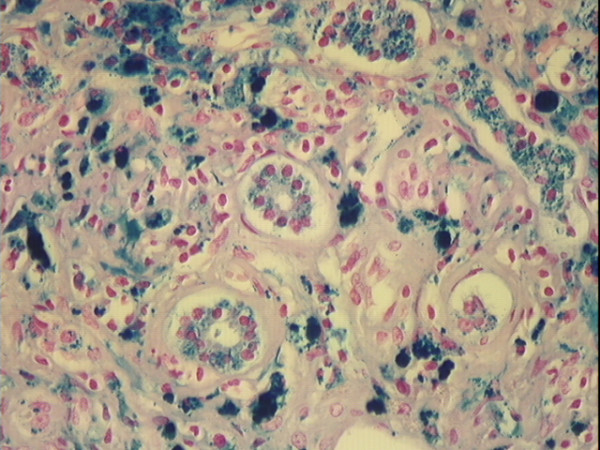
**Photomicrograph of cirrhotic liver stained with Perls' technique. **Liver of a 50 year-old African American woman with a history of chronic alcoholism. There is a predominance of iron staining (grade 4) in hepatocytes, and prominent staining of bile ductule cells. Micronodular cirrhosis and moderate-severe steatosis were also present. Original magnification 100×.

Three persons who had histories of chronic alcoholism also had hepatic cirrhosis. A 36 year-old man had grade 0 hepatocyte and grade 0 Kupffer cell iron. A 46 year-old man had grade 2 hepatocyte iron and grade 0 Kupffer cell iron. A 50 year-old woman had grade 4 hepatocyte iron and grade 0 Kupffer cell iron (Fig. 4).

### Subjects with cirrhosis

Five subjects had hepatic cirrhosis (1.5%). Three had histories of chronic alcoholism (described above). One of these three subjects, a 50 year-old woman, had grade 4 hepatocyte iron and grade 0 Kupffer cell iron (Fig. 4). Two other subjects, neither of whom had a history of alcoholism, also had hepatic cirrhosis. One was a 44 year-old man with grade 4 hepatocyte iron who died of pneumonia (Fig. 1; Table 3). The other was a 91 year-old man with grade 3 hepatocyte iron who died of intracranial hemorrhage (Table 3).

## Discussion

Heavy hepatocyte or Kupffer cell iron staining was observed in 8.8% of the present subjects. This is consistent with prevalence estimates of hepatic iron overload reported in hospital autopsy series of African Americans from other geographic areas [1,2]. The present subjects were relatively young, on the average, and approximately two-thirds died of non-natural causes. In contrast, the subjects in previous hospital autopsy series were much older, on the average, and most died of natural causes [1,2]. In one hospital autopsy series, all subjects but one were men [2]. Although there was a predominance of men in the present study, our series included 85 women. We observed that the percentages of men with increased hepatocyte or Kupffer cell iron grades were greater than those of women. This is in agreement with the greater mean iron stores of African American men than women detected in assessments of iron nutrition, and with the predominance of men in clinical and autopsy series of African Americans with primary iron overload [1,7,11,12]. Altogether, the present subjects may be more representative of African American adults in the general population than those in hospital autopsy series [1,2], although there may be fewer available observations regarding medical history in the present cases than in African Americans who died in hospital [1,2].

Microscopic estimation of liver iron content correlates well with atomic absorption spectrometry measurements in subjects in whom the histologic distribution of hepatic iron and clinical circumstances suggest hemochromatosis, *i.e.*, predominance of hepatocyte iron and no apparent explanation for iron overload [9,13-15]. These histologic criteria were pertinent to 77% of the present subjects. On the other hand, the relationship of iron grades and quantitative liver iron measurements is not well documented in subjects in whom hepatic iron deposition occurs predominantly in macrophages, like 20% of the present subjects. Hepatic iron concentrations and indices have been used as conservative, surrogate diagnostic criteria for primary iron overload in African Americans [1,2,7,16], although there has been no validation of their use in such cases. Further, some African Americans who had iron overload demonstrated by therapeutic phlebotomy had normal hepatic iron concentrations and indices [7,17]. Elevated hepatic iron indices have also been reported to occur in a variety of other conditions [18-20].

Primary iron overload in African Americans is often associated with preferential deposition of iron in macrophages in multiple organs [1,7,21]. In some cases, this is associated with the inheritance of the Q248H missense mutation of the ferroportin gene *FPN1 *[17,21,22]. Two of thirteen (15.4%) African American iron overload index patients and two of 39 (5.1%) African American control subjects who reside in central Alabama were heterozygous for *FPN1 *Q248H [21]. Nine of the present 341 (6.0%) subjects had heavy iron staining confined to Kupffer cells. Thus, *FPN1 *Q248H could account for heavy iron staining in some of the present subjects. Other African Americans with primary iron overload have a predominance of hepatocyte iron deposition. This is consistent with hemochromatosis phenotypes associated with *HFE *genotypes typical of hemochromatosis in whites (*HFE *C282Y/C282Y or C282Y/H63D) [16,21], or with common types of hemoglobinopathy or thalassemia [7,21]. Other African Americans with primary iron overload and a predominance of hepatocyte iron staining have missense mutations of the hemojuvelin gene *HJV *on Ch1q [23] or the erythroid-specific 5-aminolevulinate synthase gene *ALAS2 *on ChX [24,25]. Other putative African iron overload alleles may account for iron overload in some cases [26]. However, performing DNA analyses to detect mutations of iron-associated genes was beyond the scope of the present work.

Acquired disorders account for increased hepatic iron deposition in some African Americans. In the present study, we did not observe any subject who had heavy liver iron staining and fibrosis or cirrhosis who did not also have hepatic steatosis or inflammation. Chronic viral hepatitis C occurs in approximately 1.8% of the overall U.S. population [27], and the prevalence of chronic hepatitis C is significantly greater in African Americans than whites in the U.S. [27,28]. A greater proportion of African Americans than persons of other races respond to chronic hepatitis C infection with an increase in iron stores, after adjustment for age, alcohol intake, gender, menopausal status, education, body mass index, and poverty index [29]. More than half of the present subjects who had heavy iron staining had hepatic inflammation. It is plausible that some of these had viral hepatitis C, although this is unproven. More than one-quarter of the present subjects who had heavy iron staining also had hepatic steatosis. The prevalence of non-alcoholic steatosis and steatohepatitis is lower in African Americans than in whites [30,31], although some risk factors for non-alcoholic hepatic steatosis and steatohepatitis (obesity, insulin resistance, and diabetes mellitus) are significantly greater in African Americans than in whites in the U.S. [30-32]. Taken together, these findings suggest the development of hepatic fibrosis or cirrhosis in African Americans who have heavy hepatic iron deposition may require the synergistic effects of hepatic steatosis or inflammation.

Iron overload sometimes develops spontaneously or after repeated erythrocyte transfusion in African Americans with heritable or acquired anemia, or with myelodysplasia or acute leukemia [7,15,33-41]. Although there were no reports of heritable or acquired anemia or of multiple erythrocyte transfusions in the present subjects, such circumstances were frequent in hospital autopsy series of African Americans [1,2].

Three of the five present subjects with micronodular cirrhosis had a history of chronic alcoholism. African Americans are at greater risk than whites for developing several alcohol-related conditions, including hepatic cirrhosis [42,43]. In the present study, however, the prevalence of heavy liver iron staining was similar in subjects with and without histories of chronic alcoholism.

## Conclusions

We conclude that heavy liver iron staining is common in African American adults who were autopsied in the coroner/medical examiner office. The different histologic patterns of heavy liver iron staining we observed in the present subjects are consistent with the phenotypic and genotypic heterogeneity of primary iron overload in African Americans [21] and with the phenotypic heterogeneity of iron overload of other causes [7,29,33-41]. However, the present results do not demonstrate specific genetic or acquired causes for heavy liver iron staining in individual subjects. Further, the present results do not prove that the present subjects with heavy liver iron staining had systemic iron overload.

## Competing interests

The author(s) declare that they have no competing interests.

## Authors' contributions

JCB conceived and designed the study, reviewed and graded the liver specimens, contributed to the statistical analyses of data, and contributed to writing the manuscript. RTA reviewed and graded the liver specimens, contributed to the statistical analyses of data, and contributed to writing the manuscript. AKR reviewed and graded the liver specimens and contributed to writing the manuscript. RMB provided information on the autopsy cases, provided the liver specimens, reviewed the histology of selected liver specimens, and contributed to writing the manuscript. All authors approved of the manuscript in its final form.

## Pre-publication history

The pre-publication history for this paper can be accessed here:


